# *In Situ* Molecular Architecture of the Helicobacter pylori Cag Type IV Secretion System

**DOI:** 10.1128/mBio.00849-19

**Published:** 2019-05-14

**Authors:** Bo Hu, Pratick Khara, Liqiang Song, Aung Soe Lin, Arwen E. Frick-Cheng, M. Lorena Harvey, Timothy L. Cover, Peter J. Christie

**Affiliations:** a Department of Microbiology and Molecular Genetics, McGovern Medical School, Houston, Texas, USA; b Department of Pathology, Microbiology and Immunology, Vanderbilt University School of Medicine, Nashville, Tennessee, USA; c Department of Medicine, Vanderbilt University School of Medicine, Nashville, Tennessee, USA; d Veterans Affairs Tennessee Valley Healthcare System, Nashville, Tennessee, USA; Washington University School of Medicine; Stony Brook University; University of California, Davis

**Keywords:** *Helicobacter pylori*, cryoelectron tomography, nanomachine, pathogenesis, protein translocation, type IV secretion

## Abstract

Helicobacter pylori colonizes about half of humans worldwide, and its presence in the gastric mucosa is associated with an increased risk of gastric adenocarcinoma, gastric lymphoma, and peptic ulcer disease. H. pylori strains carrying the *cag* pathogenicity island (*cag*PAI) are associated with increased risk of disease progression. The *cag*PAI encodes the Cag type IV secretion system (Cag_T4SS_), which delivers the CagA oncoprotein and other effector molecules into human gastric epithelial cells. We visualized structures of native and mutant Cag_T4SS_ machines on the H. pylori cell envelope by cryoelectron tomography. Individual H. pylori cells contain multiple Cag_T4SS_ nanomachines, each composed of a wheel-shaped outer membrane complex (OMC) with 14-fold symmetry and an inner membrane complex (IMC) with 6-fold symmetry. CagX, CagY, and CagM are required for assembly of the OMC, whereas strains lacking Cag3 and CagT produce outer membrane complexes lacking peripheral components. The IMC, which has never been visualized in detail, is configured as six tiers in cross-section view and three concentric rings surrounding a central channel in end-on view. The IMC contains three T4SS ATPases: (i) VirB4-like CagE, arranged as a hexamer of dimers at the channel entrance; (ii) a hexamer of VirB11-like Cagα, docked at the base of the CagE hexamer; and (iii) VirD4-like Cagβ and other unspecified Cag subunits, associated with the stacked CagE/Cagα complex and forming the outermost rings. The Cag_T4SS_ and recently solved Legionella pneumophila Dot/Icm system comprise new structural prototypes for the T4SS superfamily.

## Abstract (precis)

Bacterial type IV secretion systems (T4SSs) have been phylogenetically grouped into two subfamilies. The T4ASSs, represented by the Agrobacterium tumefaciens VirB/VirD4_T4SS_, include “minimized” machines assembled from 12 VirB- and VirD4-like subunits and compositionally larger systems such as the Helicobacter pylori Cag_T4SS_. T4BSSs encompass systems closely related in subunit composition to the Legionella pneumophila Dot/Icm_T4SS_. Here, we present structures of native and mutant H. pylori Cag machines determined by *in situ* cryoelectron tomography. We identify distinct outer and inner membrane complexes and, for the first time, visualize structural contributions of all three “signature” ATPases of T4SSs at the cytoplasmic entrance of the translocation channel. Despite their evolutionary divergence, the Cag_T4SS_ aligns structurally much more closely to the Dot/Icm_T4SS_ than an available VirB/VirD4 subcomplex. Our findings highlight the diversity of T4SSs and suggest a structural classification scheme in which T4SSs are grouped as minimized VirB/VirD4-like or larger Cag-like and Dot/Icm-like systems.

## INTRODUCTION

Type IV secretion systems (T4SSs) are deployed by most species of bacteria for the conjugative transfer of mobile genetic elements (MGEs) to other bacteria and by many pathogenic species of bacteria for the interkingdom translocation of “effector” proteins to aid in infection processes (1, 2). Because of their broad medical importance, the T4SSs are excellent targets for intervention strategies aimed at suppressing antibiotic resistance spread and infection. To implement such strategies, however, it is first necessary to develop detailed knowledge of the mechanisms of action and structures of these nanomachines. Until recently, structural studies of T4SSs have advanced slowly, in large part because intact machines are refractory to isolation from the bacterial cell envelope for analysis by single-particle electron microscopy (EM). T4SSs also vary considerably in subunit composition (3, 4) (see Fig. S1A in the supplemental material). For example, in Gram-negative bacteria, “minimized” T4SSs classified as type IVA systems (T4ASSs) are assembled from 12 subunits, termed VirB1 through VirB11 and VirD4 according to a unifying nomenclature developed from the well-characterized Agrobacterium tumefaciens VirB/VirD4 system (5). However, other members of the T4ASS subfamily are assembled from these VirB/VirD4 subunits plus 10 or more system-specific components, as exemplified by the Escherichia coli F plasmid transfer system and the Helicobacter pylori Cag T4SS (referred to here as Cag_T4SS_) (6, 7). Members of a second T4SS subfamily, designated “IVB” (T4BSSs), are phylogenetically distantly related to the T4ASSs and are also compositionally more complex than the minimized systems. In the T4BSSs, as many as 20 system-specific components in addition to Vir homologs or orthologs are required for machine assembly, as represented by the Legionella pneumophila Dot/Icm system (5). These types of evolutionary adaptations impart biological diversity and likely also considerable structural variability to the members of the T4SS superfamily (4).

10.1128/mBio.00849-19.1 FIG S1 Structures of T4SSs solved to date. Download FIG S1, PDF file, 1.5 MB. Copyright © 2019 Hu et al. 2019 Hu et al. This content is distributed under the terms of the Creative Commons Attribution 4.0 International license.

Despite difficulties in isolating intact T4SSs, one intrinsically stable substructure, designated the outer membrane complex (OMC), has been recovered from different T4SSs and characterized by single-particle EM. The OMCs of minimized T4ASSs are assembled from 14 copies of three subunits (VirB7, VirB9, and VirB10) and are present as barrel-shaped structures of ∼18.5 nm in cross-section and height (8–11). Recently, OMCs from the H. pylori Cag T4ASS and the L. pneumophila Dot/Icm T4BSS were also isolated and structurally characterized (12, 13). Interestingly, the OMCs of both systems are composed of three large subunits, only certain regions of which bear sequence or predicted structural similarities to their VirB7, VirB9, and VirB10 counterparts from the minimized systems. Both OMCs are also composed of at least two system-specific subunits. Correspondingly, the Cag and Dot/Icm OMCs are considerably larger at ∼42 nm wide and architecturally more complex than those of the minimized systems.

In a remarkable advance, a nearly complete minimized T4ASS was successfully isolated and its structure was solved by single-particle EM. This large (∼3-MDa) substructure, obtained from E. coli harboring the conjugative plasmid R388, consists of an OMC connected by a thin stalk to an inner membrane complex (IMC) (14). This OMC/IMC complex is composed of homologs of the VirB3 through VirB10 subunits and is therefore termed the VirB_3–10_ complex (Fig. S1B). Two features of particular interest include the thin, connecting stalk, which lacks structural definition, and the IMC, which is highly asymmetrical. The IMC presents as an IM platform (25.5 nm wide by 10.5 nm thick) attached to two side-by-side VirB4 hexamers (10.5 nm wide by 13.5 nm long) that extend into the cytoplasm. In a recent update, one or two dimers of the VirD4 substrate receptor were shown to embed between the two VirB4 hexamers in the VirB_3–10_ subassembly (15). Although features of the IMC and central stalk leave unresolved the question of how T4SS substrates are translocated to the cell surface, significantly, the VirB_3–10_/VirD4 complex offered the first structural blueprint for a T4SS spanning the Gram-negative cell envelope.

To overcome issues of intrinsic instability and structural flexibility of T4SSs, most notably in the periplasmic regions and IMCs, we and others have capitalized on revolutionary advances in cryoelectron tomography (CryoET) to visualize T4SSs in the native context of the bacterial cell (16–18). Recently, we presented an *in situ* structure of the Dot/Icm T4BSS (17), which assembles at the poles of L. pneumophila cells (19). The wheel-shaped OMC and the nature of its association with the OM were visualized in detail, but, more importantly, we showed that the IMC differs markedly from that of the VirB_3–10_/VirD4 complex (Fig. S1B and C). The IMC of the Dot/Icm machine is highly symmetric and is dominated by two “signature” VirB ATPases, VirB4-like DotO and VirB11-like DotB, that are arranged as centrally stacked hexamers at the cytoplasmic base of the T4BSS. Furthermore, a large central cylinder connects the IMC and OMC, and a clearly visible channel extends across the entire nanomachine, thus representing the first architectural rendering of a substrate translocation route through a T4SS (17).

The H. pylori Cag machine was also recently visualized by *in situ* CryoET (18). In size and overall architecture, the Cag OMC resembles the ring-shaped complex analyzed by negative-stain EM (13). Tiers of density were shown to extend from the IM into the cytoplasm, which were proposed to constitute the IMC. In this study, we further defined the Cag_T4SS_ structure by *in situ* CryoET. Through analyses of Δ*cag* mutant machines, we visualized contributions of several subunits to the OMC and IMC. Most notably, for the first time, we visualized densities contributed by each of the three signature T4SS ATPases (VirB4-like CagE, VirB11-like Cagα, and VirD4-like Cagβ) to the IMC. Our findings enable delineation of an ordered pathway for Cag_T4SS_ machine assembly and further comparisons with other structurally solved T4SSs.

## RESULTS

### The Cag outer membrane complex.

**FIG 1:**
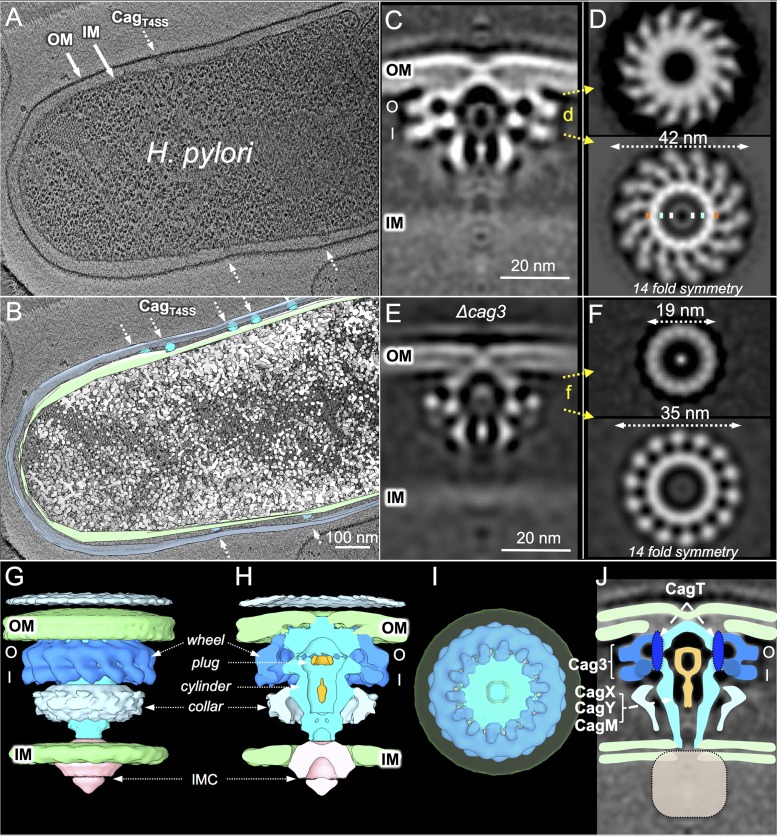
H. pylori cell with Cag T4SSs visualized by cryoelectron tomography. (A and B) Slice of a tomographic reconstruction of a typical H. pylori cell (A) and its surface rendering (B) showing multiple T4SSs (arrows) embedded in the cell envelope. OM, outer membrane; IM, inner membrane. (C) A central section of the subtomogram average structure of the intact T4SS showing the OM, IM, outer membrane complex (OMC), and central cylinder in detail. O, outer layer; I, inner layer. (D) Cross sections viewed from the top of the OMC at the positions indicated in panel C reveal the 14-fold symmetry as well as the change in chirality of the OMC across its height (see Movie S3). Colored dots denote diameters of central cylinder (19 nm, orange), central channel (14 nm, light blue), and plug domain (7 nm, white). (E and F) A central section of the subtomogram average structure of the Δ*cag3* mutant machine reveals a truncated OMC (E); cross-sectional views (F) show knobbed projections from the central cylinder with 14-fold symmetry present in the I-layer but not in the O-layer. (G, H, and I) 3D surface renderings of the Cag_T4SS_ show the O/I-layered spoked wheel and the cylinder, plug, and collar domains of the OMC in side, central cut, and top-down views. (J) A cartoon model of the OMC with proposed contributions of CagX, CagY, and CagM to the central cylinder, of CagT to the cylinder, and of Cag3 to the spoked wheel. The local refinements of the OMC did not resolve the IMC (gray box).

H. pylori strain 26695 encodes the Cag_T4SS_ and polar sheathed flagella but lacks genes encoding type IV pili or other fimbrial structures involved in virulence and DNA transformation (20). To facilitate detection of the Cag_T4SS_ nanomachines, we used a strain of 26695 that is deficient for production of flagella (21, 22). We visualized frozen-hydrated H. pylori cells by use of a direct electron detector and a high-throughput CryoET protocol applied in our previous studies to solve the structures of the Dot/Icm_T4SS_, type III secretion systems (T3SSs), and flagellar basal bodies (17, 23–25). High magnification and three-dimensional (3D) reconstruction revealed the architecture of the H. pylori cell envelope, including the inner and outer membranes, as well as multiple, cone-shaped nanomachines distributed around the envelope (Fig. 1A and B; see also Fig. S2A and Movie S1). These structures resemble the OMC subassemblies previously visualized *in vitro* and *in situ* (13, 18). An isogenic Δ*cag*PAI mutant strain lacked the cone-shaped structures, confirming the importance of the *cag*PAI for their formation (see Table S1).

10.1128/mBio.00849-19.2 FIG S2 Detection of the Cag_T4SS_ on the H. pylori cell surface and refinements of the OMC and IMC. Download FIG S2, PDF file, 0.9 MB. Copyright © 2019 Hu et al. 2019 Hu et al. This content is distributed under the terms of the Creative Commons Attribution 4.0 International license.

10.1128/mBio.00849-19.7 TABLE S1 Strains, plasmids, and CryoET data used in this study. Download Table S1, PDF file, 0.1 MB. Copyright © 2019 Hu et al. 2019 Hu et al. This content is distributed under the terms of the Creative Commons Attribution 4.0 International license.

10.1128/mBio.00849-19.8 MOVIE S1 3D visualization of a tomographic reconstruction of intact CagT4SSs in an H. pylori cell. Download Movie S1, MOV file, 4.0 MB. Copyright © 2019 Hu et al. 2019 Hu et al. This content is distributed under the terms of the Creative Commons Attribution 4.0 International license.

From 425 tomograms of different H. pylori cells, we generated 1,280 reconstructions of Cag_T4SS_ nanomachines (Table S1). After extensive subtomogram alignment and averaging, we determined the structure of the OMC at a resolution of ∼4 nm (see Fig. S2B and D), which enabled detection of structural features not previously visualized for the Cag_T4SS_ (Fig. 1C). In side view, the OMC presents as a double-layered wheel of ∼42 nm in width. The outer layer (O-layer) forms a region of continuous density across the width of the OMC that embeds into the inner leaflet and establishes contact with the outer leaflet of the OM, resulting in a distinct inward pinching of the outer leaflet. The inner layer (I-layer) extends from the outer edge of the wheel to a large central cavity or channel of ∼14 nm in width (Fig. 1D). Two densities project vertically from the bottom of the wheel and comprise a central hub with a channel that extends toward the inner membrane (IM). A key-shaped density, possibly representing a plug domain, is situated within the central channel.

The OMC has 14 spokes, all of which display a remarkable change in chirality when viewed from the OM toward the IM (Fig. 1D). At the proximal face of the I-layer, the region of the OMC closest to the IM, the spokes exhibit counterclockwise rotation. They are composed of two distinct knobs and are connected to a central cylinder of 19 nm in diameter, which corresponds to the hub (visualized in side view). A second internal ring of ∼7 nm in diameter corresponds to the putative plug domain. In striking contrast, near the OM, the spokes have a clockwise rotation and appear to consist of only one knob. The change in chirality, which is readily visualized by scanning from the distal to the proximal face of the Cag OMC (Movie S3), has not been reported previously for any T4SS. In contrast to the Cag OMC, in the recently solved L. pneumophila Dot/Icm, the spokes associated with the OMC have a clockwise rotation in top-down view, and a similar scan across the OMC does not reveal a detectable change in chirality (Movie S3). The 14-fold symmetric features of the OMC were revealed in our 3D classifications using reference-free alignment, and the architecture of the OMC was resolved further by imposing 14-fold symmetry during refinement (Fig. 1C and D; see also Fig. S2B and D). A collar of density encircles the central cylinder as it extends from the base of the OMC. Through local refinement, we were able to visualize the region of the cylinder and collar extending from the OMC to the IMC, although apparent flexibility of the collar prevented detailed reconstructions (Fig. S3A). The architecture of the OMC, composed of the outer wheel, cylinder and channel, central plug, and periplasmic collar, is displayed clearly in 3D reconstructions, which are presented as side and cutaway views (Fig. 1G to I).

10.1128/mBio.00849-19.3 FIG S3 Refinement of the native Cag_T4SS_ and visualization of Δ*cag3* and Δ*cagT* mutant machines. Download FIG S3, PDF file, 1.3 MB. Copyright © 2019 Hu et al. 2019 Hu et al. This content is distributed under the terms of the Creative Commons Attribution 4.0 International license.

### Cag subunit contributions to the OMC.

The purified Cag_T4SS_ OMC is composed of five subunits (CagX, CagY, CagM, CagT, and Cag3) (13). CagX, CagY, and CagT are orthologs of the signature VirB9, VirB10, and VirB7 subunits, although they actually exhibit very limited sequence similarities with their VirB counterparts (7). CagM and Cag3 are specific for the Cag_T4SS_ (7, 26, 27). Prior attempts to isolate OMCs from strains deleted of CagX, CagY, and CagM were unsuccessful, but definitive conclusions regarding the contributions of these subunits to OMC assembly were not possible due to the indirect methods used for machine isolation (13). Here, we confirmed by *in situ* CryoET that Δ*cagX*, Δ*cagY*, and Δ*cagM* mutant cells lack detectable Cag_T4SS_ machines (Table S1). CagX, CagY, and CagM thus are essential for assembly of the central cylinder and, in turn, of the entire Cag_T4SS_.

It was also previously shown that OMCs recovered from strains deleted of Cag3 or CagT lack the outer spoked wheel and instead consist only of central rings with an estimated diameter of 19 nm, the size of the central cylinder identified here by *in situ* CryoET (13). We refined the structure of the Δ*cag3* mutant machine by analysis of 172 machine particles from 367 tomograms (Fig. 1E and F; see also Fig. S3B and Table S1). The O-layer of the OMC visualized in side view appears to be missing the entire peripheral densities, whereas the I-layer lacks only the outer set of knobs of the spoked wheel. As a result, the Δ*cag3* OMC has a larger cross-section (35 nm) than previously reported (19 nm) (13). These findings indicate that Cag3 is essential for assembly of most of the peripheral spoked-wheel structure but not for recruitment of a subunit(s) comprising the inner set of knobs in the I-layer (Fig. 1J).

Δ*cagT* mutant complexes, as visualized previously by negative staining EM (nsEM) (13), are composed of only the central rings, but the rings are notably thinner than those associated with the native OMC. Here, we determined that the Δ*cagT* mutant machines (400 tomograms) have central rings and lack peripheral densities (Table S1). However, the Δ*cagT* mutant machines were highly structurally variable and were rarely found on the cell surface (<1 machine per 10 cells), which prevented structural refinements (Fig. S3B). Our findings together, with those from the previous nsEM analyses, thus support a general assignment for CagT at the periphery of the central cylinder, where it plays a stabilizing role for the central cylinder and also mediates formation of the entire peripheral spoked wheel (Fig. 1J).

### Visualization of the ATPase energy center.

**FIG 2:**
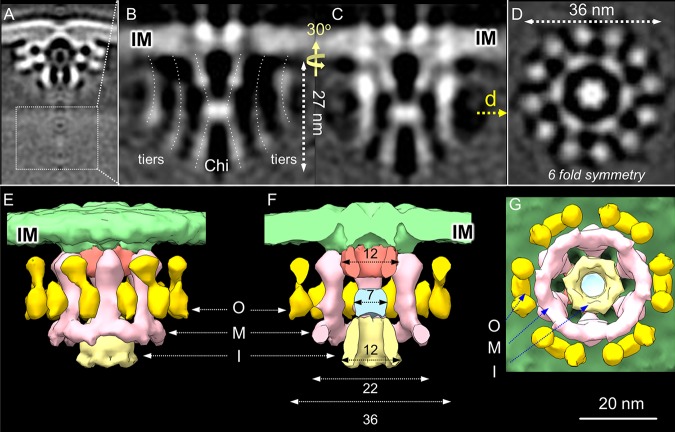
The Cag_T4SS_ IMC. (A) A local refinement of the cytoplasmic portion of the Cag_T4SS_ (boxed) revealed a detailed structure of the IMC. (B) Central section of the subtomogram average structure of the IMC. IM, inner membrane. The central Chi (X) structure and flanking tiers are identified (dashed lines). (C) The section shown in panel B presented in a different view as indicated by the vertical and curved arrows shown between panels B and C. (D) Cross section of the IMC at the position indicated in panel C revealing the 3-ring architecture and 6-fold symmetry of the IMC. (E, F, and G) 3D surface renderings of the T4SS machine IMC presented in side (E), central cut (F), and bottom (G) views. I, inner ring; M, middle ring; O, outer ring. Numbers correspond to the diameters (in nanometers) of the upper, middle, and lower regions of the central Chi and of the M and O rings. Panel G shows the hexameric arrangements of the (i) bottom portion of the central Chi (I-ring), (ii) M ring with the 6 tiers joined at their bases, and (iii) O ring composed of 12 tiers arranged in 6 pairs.

We locally refined the cytoplasmic region from the 1,280 Cag_T4SS_ particle reconstructions to solve the structure of the IMC in unprecedented detail (Fig. 2A to C; see also Table S1). In side view, the IMC is composed of a central Chi (X) structure that attaches to the IM and extends 27 nm into the cytoplasm. The Chi structure is flanked by two sets of vertical tiers that also project into the cytoplasm. In end-on view, the IMC presents as three concentric hexameric rings (Fig. 2D). The Chi structure, which forms the inner (I) ring, varies in diameter along its length from 12 nm at the two termini to 7 nm at the center (Fig. 2F). The middle (M) ring of 22 nm in diameter is composed of 6 tiers whose bases are joined to form a continuous ring (Fig. 2D to G). The outer (O) ring of 36 nm in diameter is composed of 12 tiers grouped in 6 pairs (Fig. 2D to G). Both the 6-fold symmetry and 3-ringed architecture of the IMC are clearly visible in the initial 3D classifications (Fig. S2C). This 6-fold symmetry was imposed during refinement, resulting in a structure for the IMC revealed at ∼4.7-nm resolution (Fig. S2D). 3D surface renderings clearly show the distinct 3-ring architecture of the IMC in side, central cut, and bottom views (Fig. 2E to G; see also Movie S2). Finally, the cytoplasmic densities appear to connect to densities that span the IM, which might correspond to an IM platform analogous to that described previously for the VirB_3–10_ substructure (14).

10.1128/mBio.00849-19.9 MOVIE S2 3D visualization of the entire CagT4SS showing architectural features of the OMC and IMC. Download Movie S2, MOV file, 13.4 MB. Copyright © 2019 Hu et al. 2019 Hu et al. This content is distributed under the terms of the Creative Commons Attribution 4.0 International license.

### Contributions of the Cagβ, Cagα, and CagE ATPases to IMC assembly.

The 3-ringed architecture of the IMC is unprecedented among the T4SS nanomachines characterized to date (14, 16–18). Previous studies have suggested that a number of IM-associated Cag subunits contribute to Cag_T4SS_ function (27, 28). These include the three signature ATPases (VirB4-like CagE, VirB11-like Cagα, and VirD4-like Cagβ), two VirB-like integral IM proteins (VirB6-like CagW and VirB8-like CagV), and several IM or cytoplasmic subunits specific to the Cag system (e.g., CagF, CagH, CagZ, and CagU). To begin deciphering the contributions of individual Cag subunits to IMC assembly, here we analyzed structures of mutant machines produced by strains deleted of the ATPases.

**FIG 3:**
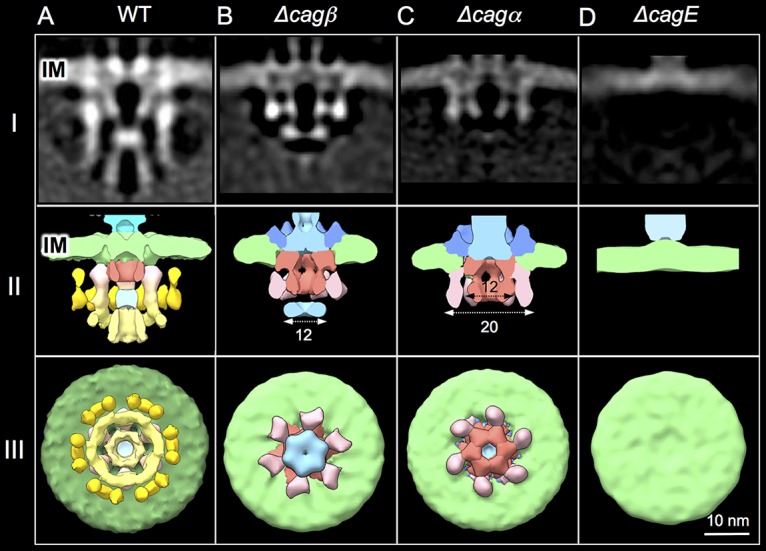
Contributions of the Cag ATPases to the Cag_T4SS_ IMC. Columns A to D present structures of the IMCs from the native (WT) and Δ*cag*β, Δ*cag*α, and Δ*cagE* mutant machines. (Row I) Central slices of the averaged IMC structures. IM, inner membrane. (Row II) 3D surface renderings, cutaway side views. Numbers correspond to diameters (in nanometers) of the regions shown. (Row III) 3D surface renderings, bottom views. The lower regions of the I and M rings are shaded in yellow and, together with the yellow-shaded O-ring, reflect density contributions to the IMC associated with production of Cagβ.

First, we defined the structure of the Δ*cagβ* mutant machine by subtomogram averaging of 2,278 machine particles from 666 tomograms. Strikingly, in mutant machines lacking VirD4-like Cagβ, the OMC is present (Fig. S3C) but several densities are missing in the IMC compared with that of the native machine. These include the basal arms of the Chi structure, the basal halves of the first set of flanking tiers, and the entire second set of flanking tiers (Fig. 3A and B, rows I and II). Consequently, the IMC of the Δ*cagβ* mutant is honed mainly to a set of side-by-side inverted “V” structures extending from the IM into the cytoplasm. The inner arms of the V structure correspond to the upper half of the Chi structure and the outer arms to the upper half of the first set of flanking tiers. A disc is located at the base of the inner arms of the V structures, which corresponds to the center of Chi structure. Interestingly, this central disc (∼12 nm) is somewhat wider than the corresponding disc in the wild-type (WT) machine (∼7 nm) (see Discussion). In end-on view, the Δ*cagβ* mutant machine is missing the entire O-ring composed of 6 pairs of tiers. The M-ring and I-ring are present but differ in diameter and architecture from those in the native complex; e.g., the M-ring presents as 6 knobs as opposed to a continuous ring (Fig. 3B, row III). The most important outcome from our comparative analyses of the WT and Δ*cagβ* mutant machines is that, for the first time, we have been able to identify IMC densities associated with production of a VirD4-like homolog using *in situ* CryoET.

Next, we determined the effect of deleting VirB11-like Cagα. Analyses of 1,135 machine particles from 419 tomograms showed that the Δ*cagα* mutant machines are missing not only the densities accompanying deletion of Cagβ but also the central disc corresponding to the axis of the Chi structure in the native complex (Fig. 3C, rows I and II). In end-on view, the central disc is missing, but the six inner arms of the inverted V structures are connected to form a central hexameric ring and the six outer arms splay outward to form a knobbed ring structure (Fig. 3C, row III). Therefore, the only detectable IMC densities in the Δ*cagα* mutant are the side-by-side V densities whose apices embed into the IM. These findings allow provisional assignment of Cagα to the central disc of the Chi structure, with the assignment gaining support from our recent studies of the L. pneumophila Dot/Icm system (see Discussion).

Finally, we determined the structure of the Δ*cagE* mutant machine by subtomogram averaging of 465 machine particles from 242 tomograms (Fig. 3D). As observed for the Δ*cagβ* and Δ*cagα* mutations, the Δ*cagE* mutation had no effect on assembly of the OMC (Fig. S3C). Strikingly, however, the Δ*cagE* mutant machine completely lacks the entire cytoplasmic complex, including the V structures detected in the Δ*cagα* and Δ*cagβ* mutant machines (Fig. 3D, rows I to III). Production of CagE is therefore critical for assembly of the entire cytoplasmic portion of the IMC. We propose that CagE contributes to the V-shaped densities, an assignment that also gains support from our recent analyses of the Dot/Icm machine (see Discussion).

## DISCUSSION

**FIG 4:**
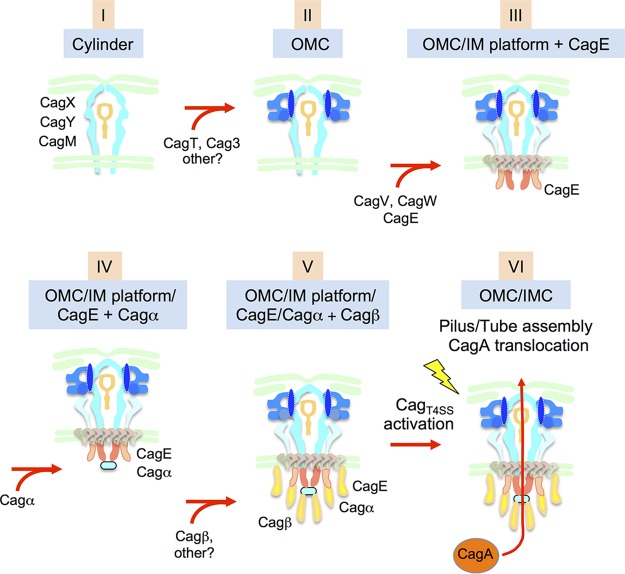
Model depicting an outside-to-inside assembly pathway for the Cag_T4SS_. The central cylinder assembles first and serves as a scaffold for elaboration of the OMC and then the IMC. Our findings highlight the sequential order of assembly of the Cag ATPases as follows: CagE is initially recruited followed by Cagα and, finally, Cagβ. Cag subunits in addition to those shown are recruited to build out the OMC and IMC. In H. pylori cells exposed to human epithelial cells, the Cag_T4SS_ is activated (yellow lightning bolt) to assemble extracellular pili or sheathed tubes and translocate the CagA substrate. Structural changes accompanying Cag_T4SS_ activation are not yet defined. See text for further details.

We solved structures of native and mutant Cag_T4SS_ nanomachines in the natural context of the H. pylori cell envelope by *in situ* CryoET. The 3D structure of the native machine revealed new details about the Cag OMC, and analyses of mutant machines supplied new insights into the locations and roles of OMC subunits to machine assembly and architecture. Most importantly, we visualized the Cag IMC, whose 6-fold symmetrical, 3-ringed configuration is the most structurally complex of T4SS IMCs solved to date. We also identified contributions by each of the three signature T4SS ATPases to IMC assembly. Results of our *in situ* CryoET studies, combined with previous biochemical and structural findings, support a new model for the out-to-in assembly of the Cag_T4SS_ across the H. pylori cell envelope (Fig. 4). This biogenesis pathway forms a framework for the following discussion of distinguishing structural features of the Cag_T4SS_.

### The Cag_T4SS_ cylinder/OMC scaffold.

In our working model, the Cag OMC is built sequentially, by elaboration first of the Cag cylinder and then the outer spoked wheel. In step I, the three “core” subunits CagX, CagY, and CagM assemble as the Cag cylinder, a substructure sufficiently stable for isolation from cells and structural characterization by single-particle EM (13). Here, we confirmed that the Cag cylinder is visible even in the absence of OMC subunits CagT and Cag3 (Fig. 1E and F; see also Fig. S3B) and that the entire OMC is detectable in mutants deleted of the ATPases (see Fig. S3C). The capacity of the Cag cylinder to assemble independently of other Cag subunits is reminiscent of earlier findings for the OMCs of the A. tumefaciens VirB/VirD4 system and other minimized T4ASSs (8, 11, 29, 30). In fact, the Cag cylinder exhibits several structural features that are strikingly similar to those of the OMCs of minimized T4ASSs, including a double layer (O/I) architecture, ∼18.5 nm width, and 14-fold symmetry (8). This similarity extends to the level of subunit composition, in which orthologs of VirB9 and VirB10 elaborate both the Cag cylinder and OMCs of minimized systems. In the minimized systems, VirB9 (30 kDa) and VirB10 (48 kDa) form an extensive network of contacts that, together with the small (4.5-kDa) VirB7 lipoprotein, comprise an intrinsically stable OMC and nucleation scaffold for the rest of the T4SS machine. Furthermore, an α-helical domain (“antenna projection” [AP]) near the C terminus of VirB10 assembles as a pore across the OM (8–10, 31). In the Cag_T4SS_, the extreme C termini of the much larger VirB9-like CagX (55-kDa) and VirB10-like CagY (180-kDa to 220-kDa) subunits adopt the respective VirB structural folds, including the VirB10 AP, as shown by an available X-ray structure for a portion of CagX (32) and by Phyre2 modeling (Fig. S4). These motifs of CagX and CagY can thus be predicted to form the portion of the Cag cylinder corresponding to the OMCs of minimized systems, which embeds into and spans the H. pylori OM.

10.1128/mBio.00849-19.4 FIG S4 Sequence and structural comparisons of VirB7, VirB9, and VirB10 components. Download FIG S4, PDF file, 0.2 MB. Copyright © 2019 Hu et al. 2019 Hu et al. This content is distributed under the terms of the Creative Commons Attribution 4.0 International license.

In the minimized T4ASSs, VirB7 lipoproteins stabilize the OMC and anchor it to the inner leaflet of the OM. Although Cag cylinders can form in the absence of the VirB7-like CagT lipoprotein, they are nonabundant and morphologically variable, as shown by *in situ* CryoET analyses (Fig. 1E and F; see also Fig. S3B). They are also thinner than WT cylinders when isolated from Δ*cagT* mutant cells and analyzed by single-particle EM (13). These findings suggest that CagT plays a stabilizing function for the Cag cylinder that is more VirB7-like than previously envisioned (Fig. 4, step II). Interestingly, only the N terminus of CagT (29 kDa) is related to the comparatively smaller VirB7 subunits of minimized systems. The large C-terminal region lacks a structural homolog in the protein structure databases. However, C termini of other large VirB7-like lipoproteins, such as the 13-kDa VirB7_Xc_ and 16-kDa DotD associated with a Xanthomonas citri T4ASS and the L. pneumophila Dot/Icm T4BSS, respectively, have been shown to adopt globular N0 domains at their C termini (Fig. S4). Recently, Farah and Waksman and their colleagues determined that the N0 domains of VirB7_Xc_ subunits splay away from the central portion of the *X. citri* OMC, giving rise to a set of 14 peripheral knobs (11). This structural arrangement is reminiscent of the 14-knob pattern visualized for the Δ*cag3* mutant machine (Fig. 1F), which is not detected in the Δ*cagT* mutant. CagT thus might adopt an overall architecture similar to that described for VirB7_Xc_, in which the N-terminal VirB7-like region lines the periphery and stabilizes the central cylinder and the C-terminal region adopts a globular fold comprising the inner knobs of the outer spoked wheel (Fig. 4, step II). These C-terminal knobs are excellently positioned to recruit Cag3, which has the intrinsic ability to oligomerize as large ring-shaped complexes *in vitro* (33), to build out the OMC. Cag3 is fairly large (∼55 kDa) and yet is still unlikely to account for the entirety of the OMC densities missing in the Δ*cag3* mutant, suggesting that other Cag subunits are also recruited to complete OMC assembly.

As the Cag cylinder extends from the OMC through the periplasm to the IM, it maintains an overall ∼19-nm diameter and central channel (Fig. 2). This contrasts with the VirB_3–10_ substructure, in which the OMC abruptly narrows and connects to a thin, flexible stalk that extends to the IMC (14) (Fig. S1B). In all T4SSs characterized to date, VirB10-like subunits have been shown to span the entire cell envelope, which forms a basis for their functions as structural scaffolds during machine biogenesis and as sensors and transducers of intracellular and extracellular signals for machine activation (34–37). VirB10 subunits are therefore thought to comprise part of the stalks or cylinders connecting the IMC and OMC subassemblies (8–11, 14, 31, 38). In the minimized systems, the periplasmic linker domains of VirB10 subunits are fairly small (∼125 residues) and rich in Pro residues, features potentially contributing to the stalk’s flexibility (Fig. S4) (11, 31, 38). The corresponding region of CagY, by contrast, is exceptionally large (>1,300 residues) and designated as the multiple-repeat region (MRR) because it is composed of many direct repeat sequences (Fig. S4) (39). Detailed studies have shown that CagY subunits undergo extensive intragenic recombination events between the DNA repeat sequences, resulting in many duplications and insertions that affect Cag_T4SS_ function, as monitored by CagA translocation and interleukin-8 (IL-8) induction during H. pylori infection of epithelial cells (39, 40). In view of our finding that the Cag cylinder extends the length of the periplasm and houses a central channel, it is tempting to suggest that the observed host-induced sequence variability within CagY’s MRR serves to fine-tune Cag_T4SS_ function through modulation of the Cag cylinder/channel architecture.

### Comparison of the Cag and Dot/Icm OMCs.

The shared features of the Cag_T4SS_ cylinder and OMCs of minimized T4SSs described above provide insights into the evolutionary trajectory of the Cag_T4SS_. Furthermore, by having solved the *in situ* structures of both the Cag and Dot/Icm systems, we are uniquely positioned to compare the architectures of the two systems in their native cellular contexts. The Dot/Icm system, for example, closely resembles the Cag_T4SS_ insofar as its OMC presents as an ∼42-nm outer-spoked wheel with a central cylinder, which extends to the IMC surrounded by a collar (Fig. S5) (17). Furthermore, like the Cag system, the Dot/Icm machine is composed of 5 subunits, among which DotD, DotH, and DotG are much larger than their VirB7, VirB9, and VirB10 counterparts and 2 subunits (DotF and DotC) are system specific (12). One interesting difference between the two OMCs, the basis of which will be better understood when higher-resolution structures are available, is that the OMC of the Dot/Icm system has a consistent clockwise rotation whereas that of the Cag machine changes its chirality across its height (see Movie S3 in the supplemental material). Despite this difference, the distantly related Cag and Dot/Icm systems clearly have evolved their OMCs by assembling a central cylinder that corresponds to the minimized VirB7/B9/B10 OMCs and then building out the subcomplex through exaptation of domains and subunits from undefined ancestries. At this time, it is not immediately obvious why some T4SSs have built structural complexity into their OMCs, especially insofar as many minimized systems function efficiently to deliver DNA between bacteria or effector proteins to eukaryotic target cells. Perhaps large OMCs contribute directly to establishment of target cell contacts or indirectly to target cell binding through their roles in assembly of associated conjugative pili or other attachment organelles (see below) (6, 18, 41–44).

10.1128/mBio.00849-19.10 MOVIE S3 Scans showing the change in chirality of the OMC from the Cag_T4SS_ system but not the OMC from the Dot/Icm system. Download Movie S3, MOV file, 0.7 MB. Copyright © 2019 Hu et al. 2019 Hu et al. This content is distributed under the terms of the Creative Commons Attribution 4.0 International license.

10.1128/mBio.00849-19.5 FIG S5 Outer membrane complexes of the H. pylori Cag and L. pneumophila Dot/Icm T4SSs. Download FIG S5, PDF file, 1.2 MB. Copyright © 2019 Hu et al. 2019 Hu et al. This content is distributed under the terms of the Creative Commons Attribution 4.0 International license.

### The Cag_T4SS_ IMC: toward a structural definition of the entire T4SS ATPase energy center.

In the next steps of Cag_T4SS_ machine assembly, the OMC/cylinder forms a scaffold for elaboration of the IM platform composed minimally of VirB6-like CagW and VirB8-like CagV and, in turn, recruitment of the Cag ATPases (Fig. 4, steps III to V). The structure of the Cag machine within the IM is difficult to resolve due to background noise of IM phospholipids and proteins in our CryoET tomograms. We are therefore unable to assign a temporal order to IM platform assembly versus CagE recruitment at this time. However, our studies of the Cag ATPase mutant machines clearly support the notion that an ordered pathway exists in which VirB4-like CagE is recruited first (step III), followed by docking of VirB11-like Cagα (step IV) and, finally, the VirD4-like Cagβ substrate receptor (step V). Independently of any specific positional assignments of the ATPases to the IMC, this temporal order of IMC assembly is rationalized on the basis of observed effects of the ATPase deletions on the IMC densities (Fig. 3).

Our data are also consistent with the proposal that CagE comprises the central V structures and that Cagα forms the central disc at the base of CagE. Existence of such a stacked architecture for these two ATPases gains further support from our finding that the Cag IMC bears features very similar to those that we previously described for the IMC of the Dot/Icm system (Fig. S6) (17). For example, both systems possess inverted V structures and a central disc docked at the base of the inner arms of the V structures. Deletions of the respective VirD4, VirB11, and VirB4 homologs also confer strikingly similar effects on the Cag and Dot/Icm IMCs as follows: (i) deletions of the VirB4 homologs abolish all detectable cytoplasmic densities; (ii) deletions of the VirB11 homologs yield only the inverted V structures; and (iii) deletions of the VirD4 homologs yield only the inverted V’s and central disc (Fig. S6). In the Dot/Icm system, the addition of an mCherry tag to the C terminus of VirB4-like DotO blocked the ability of DotB to dock onto the IMC. We also detected a green fluorescent protein (GFP) tag attached to the C terminus of VirB11-like DotB as an extra density on the cytoplasmic face of the central disc by *in situ* CryoET. These findings supported the conclusion that DotO assembles as a hexamer of dimers, which accounts for the V densities observed in the side view and the ring densities in the end-on view, and that a hexamer of DotB docked onto the inner ring of DotO accounts for the central disc (17).

10.1128/mBio.00849-19.6 FIG S6 Cytoplasmic complexes of the L. pneumophila Dot/Icm and H. pylori Cag T4SSs. Download FIG S6, PDF file, 1.4 MB. Copyright © 2019 Hu et al. 2019 Hu et al. This content is distributed under the terms of the Creative Commons Attribution 4.0 International license.

Despite the striking similarities of the Cag and Dot/Icm IMCs with respect to the hexameric configuration of the VirB4- and VirB11-like ATPases, the Cag IMC has at least two distinctive features. First, the central disc positioned at the base of CagE, which we provisionally assign as Cagα, is narrower in the native Cag_T4SS_ (∼7 nm) than in the Δ*cagβ* mutant (∼12 nm) (Fig. 3). This finding is of interest in view of crystallographic evidence that the Cagα hexamer has a diameter of ∼12 nm (45, 46). The different dimensions of the central disc might reflect altered conformations of Cagα in different genetic contexts, e.g., native versus Δ*cagβ* mutant, which is consistent with reports that Cagα and other members of the AAA ATPase superfamily undergo profound conformational changes as a function of nucleotide binding and hydrolysis (45–47). Another explanation derives from an earlier report that a novel regulatory protein, HP1451, interacts with and induces a closed, catalytically dead conformation of Cagα. This finding led to a proposal that HP1451 cycles on and off Cagα to regulate its ATPase activity and, in turn, substrate flow through the Cag_T4SS_ (48). The different dimensions of the central disc might reflect distinct states of Cagα in the absence or presence of bound HP1451. Finally, it is interesting to note that, in the Dot/Icm system, VirB11-like DotB dynamically cycles on and off the Dot/Icm T4SS by a mechanism dependent on ATP binding and hydrolysis (17). Indeed, this finding necessitated the use of a catalytically dead variant (DotB.E191K) to detect DotB’s stable association with DotO by *in situ* CryoET. Conceivably, in the Cag_T4SS_, Cagα also dynamically associates with the IMC in certain genetic contexts, e.g., the native system, but stably associates with the IMC in other contexts, e.g., the Δ*cagβ* mutant. Our ongoing studies include attempts to confirm the location of Cagα and establish if it stably or dynamically associates with the IMC.

Second, although we were able for the first time to visualize changes in the architecture of a T4SS IMC accompanying deletion of a VirD4-like substrate receptor by *in situ* CryoET (Fig. 3), we cannot yet assign Cagβ to any of the peripheral tiers of density that are missing in the Δ*cagβ* mutant. In fact, such an assignment is complicated by the assumption that VirD4-like substrate receptors assemble as large homohexamers *in vivo*, based on an early X-ray structure and results of phylogenetic studies showing that the VirD4 ATPases share an ancestry with the DNA motor proteins SpoIIIE and FtsK, both of which assemble as homohexamers (49, 50). If Cagβ assembles as a homohexamer, it might comprise the inverted hexameric “cup” by docking onto Cagα in the I-ring (Fig. 3, light yellow). The TrwB structural prototype for the VirD4 ATPases has a diameter of ∼12 nm, which fits well with the dimension of this cup (49, 50). Alternatively, a Cagβ hexamer might form through the docking of monomers onto the outer arms of CagE to yield the M-ring (Fig. 3, darker yellow). Notably, these monomers connect at the proximal ends to yield the contiguous hexameric M-ring. However, there is also biochemical and structural evidence indicating that VirD4 subunits might adopt different oligomeric states *in vivo* (15, 51, 52), which leaves open the possibility that Cagβ could comprise the dimer pairs forming the O-ring of the Cag IMC (Fig. 3, darkest yellow). The soluble domain of Cagβ is only ∼55 kDa. Consequently, regardless of its oligomeric state, the presence of Cagβ cannot account for all of the densities missing in the Δ*cagβ* mutant. The fully assembled ATPase energy center thus likely recruits other cytoplasmic Cag subunits to build out the IMC.

### Final stage assembly: activation of the Cag_T4SS_.

The Cag_T4SS_ is activated for CagA translocation only in the presence of epithelial cells (28), which implies that the Cag_T4SS_ machines analyzed in our study correspond to quiescent structures. We postulate that, in the final step of assembly, the Cag_T4SS_ is activated to translocate CagA and other substrates into host cells (Fig. 4, step VI). This might occur through direct contact between the surface-exposed portion of the Cag_T4SS_ and epithelial cells. However, H. pylori also has been reported to elaborate two *cag*PAI-dependent extracellular organelles under conditions of growth in the presence of epithelial cells (22, 53–55). One type, termed the pilus, has diameters of ∼8 to ∼9 nm, approximating the width of conjugative pili elaborated by dedicated conjugation machines in Gram-negative species (41, 42, 54, 56). The second type, termed “sheathed tubes,” have much larger diameters of ≥37 nm (18, 22, 53). Recently, sheathed tubes were visualized by *in situ* CryoET. Remarkably, these tubes correspond to extrusions of the OM, such that the walls are composed of OM phospholipids and a surrounding sheath of extracellular lipopolysaccharides (LPSs) (18). The sheathed tubes were proposed to form by nucleation of Cag pilus-like fibers in the periplasm, which are then enveloped by the OM during outgrowth. An interesting feature of the sheathed tubes is that they are also partly composed of the large core subunit CagY, leading to a proposal that the Cag_T4SS_ undergoes profound structural rearrangements upon exposure of H. pylori cells to epithelial cells (22, 53). Clearly, the next steps in structure-function analyses of the H. pylori Cag system are to define physical relationships among the Cag_T4SS_, pili, and sheathed tubes, to visualize Cag_T4SS_ structural rearrangements accompanying machine activation by host-derived signals, and to identify specific contributions of the various *cag*PAI-encoded organelles to Cag_T4SS_ translocation.

## MATERIALS AND METHODS

### Bacterial strains and growth conditions.

H. pylori 26695 and isogenic *cag* mutant strains were grown on Trypticase soy agar plates supplemented with 5% sheep blood or on *Brucella* agar plates supplemented with 5% fetal bovine serum at 37°C in room air containing 5% CO_2_ (13). H. pylori mutant strains were selected based on resistance to chloramphenicol (5 μg/ml), kanamycin (10 μg/ml), or metronidazole (7.5 to 15 μg/ml). E. coli strain DH5α, used for plasmid propagation, was grown on Luria-Bertani (LB) agar plates or in LB liquid medium supplemented with ampicillin (50 μg/ml), chloramphenicol (25 μg/ml), or kanamycin (25 μg/ml), as appropriate. All mutant strains were generated from H. pylori strain 26695, which harbors a functional Cag_T4SS_. A Δ*cag* PAI mutant was described previously (41, 57). Unmarked Δ*cagX*, Δ*cag3*, Δ*cagM*, Δ*cagT*, and Δ*cagY* mutant strains were generated previously using counterselection methods (41, 42). Specifically, the Δ*cag3*, Δ*cagM*, Δ*cagT*, and Δ*cagY* mutants were generated using Δ*rdxA* and a *cat-rdxA* cassette as selectable markers, and the Δ*cagX* mutant was generated using *rpsL*-Lys43Arg and a *cat-rpsL* cassette as selectable markers (13, 42). For the current study, strains containing unmarked deletion mutations in *cagα*, *cagβ*, or *cagE* (Δ*cagα*, Δ*cagβ* , or Δ*cagE* mutants) were generated using *ΔrdxA* and a *cat-rdxA* cassette as selectable markers.

### Preparation of frozen-hydrated specimens.

Bacterial cultures were grown 48 h on Trypticase soy agar plates supplemented with 5% sheep blood in room air supplemented with 5% CO_2_. Bacteria were removed from the plates and suspended in phosphate-buffered saline (PBS) and were then mixed with 10-nm-diameter colloidal gold particles (used as fiducial markers in image alignment) and deposited onto freshly glow-discharged, holey carbon grids for 1 min. The grids were blotted with filter paper and rapidly frozen in liquid ethane, using a gravity-driven plunger apparatus as previously described (25, 58).

### Cryo-ET data collection and 3D reconstructions.

The frozen-hydrated specimens were imaged at −170°C using a Polara G2 electron microscope (FEI Company) equipped with a field emission gun and a direct detection device (Gatan K2 Summit). The microscope was operated at 300 kV at a magnification of −9,400, resulting in an effective pixel size of 4.5 Å at the specimen level as previously described (17). We used the tomographic package SerialEM (59) to collect low-dose, single-axis tilt series with dose fractionation mode with defocus at about 6 μm and a cumulative dose of ∼60 e^−^/Å^2^ distributed over 35 stacks. Each stack contains ∼8 images. Over 2,600 tilt series were collected at angles from −51° to 51° with increment of 3°. We used Tomoauto (58) to facilitate data processing, which included drift correction of dose-fractionated data using Motioncorr (60) and assembly of corrected sums into tilt series, automatic fiducial seed model generation, alignment and contrast transfer function correction of tilt series by IMOD (61), and reconstruction of tilt series into tomograms by Tomo3D (62). Each tomographic reconstruction was 3,710 by 3,838 by 2,400 pixels and ∼130 Gb in size. In total, 2,674 tomographic reconstructions from 10 different strains were generated (see Table S1).

### Subtomogram averaging and correspondence analysis.

We used tomographic package I3 (0.9.9.3) for subtomogram analysis as described previously (63). A total of 5,525 Cag secretion machines (400 × 400 × 400 voxels) were visually identified and then extracted from 1,924 cryo-tomographic reconstructions. Two of the three Euler angles of each Cag secretion machine were estimated based on the orientation of each particle in the cell envelope. To accelerate image analysis, 4 × 4 × 4 binned subtomograms (100 × 100 × 100 voxels) were used for initial alignment. The alignment proceeded iteratively, with each iteration consisting of three parts in which references and classification masks are generated, subtomograms are aligned and classified, and, finally, class averages are aligned to each other. Classification focusing on the OMC displayed 14-fold symmetry; therefore, 14-fold symmetry was imposed in the following processing to assist the initial alignment process. Class average structures with similar features were selected together for an iterative cycle of alignment and classification until each conformation consists of highly homogeneous particles. Further classification focusing on the cytoplasmic complex showed a hexagonal structure in four different classes. After multiple cycles of alignment and classification for 4 × 4 × 4 binned subtomograms, we used 2 × 4 × 2 binned subtomograms for refinement. Fourier shell correlation (FSC) between the two independent reconstructions was used to estimate the resolution of the averaged structures (see Fig. S2D).

### 3D visualization.

We used IMOD to visualize the maps and also to generate 3D surface rendering of H. pylori cell and UCSF Chimera (64) to visualize subtomogram averages in 3D and molecular modeling. Video clips were generated for the supplemental videos using UCSF ChimeraX and were edited with iMovie.

### Data availability.

Density maps and coordinate data that support the Cag_T4SS_ structure determined by cryo-electron tomography have been deposited in EMDB (EMD-0634 and EMD-0635). We declare that all other data supporting the findings of this study are available within the paper and its supplemental information files.
