# Supplementary material for: In Situ Molecular Architecture of the Helicobacter pylori Cag Type IV Secretion System
Source: mBio. 2019 May 14;10(3):e00849-19. doi: 10.1128/mBio.00849-19 (PMC6520456; doi:10.1128/mBio.00849-19)
Supplement: FIG S1 [file mBio.00849-19-sf001.pdf]

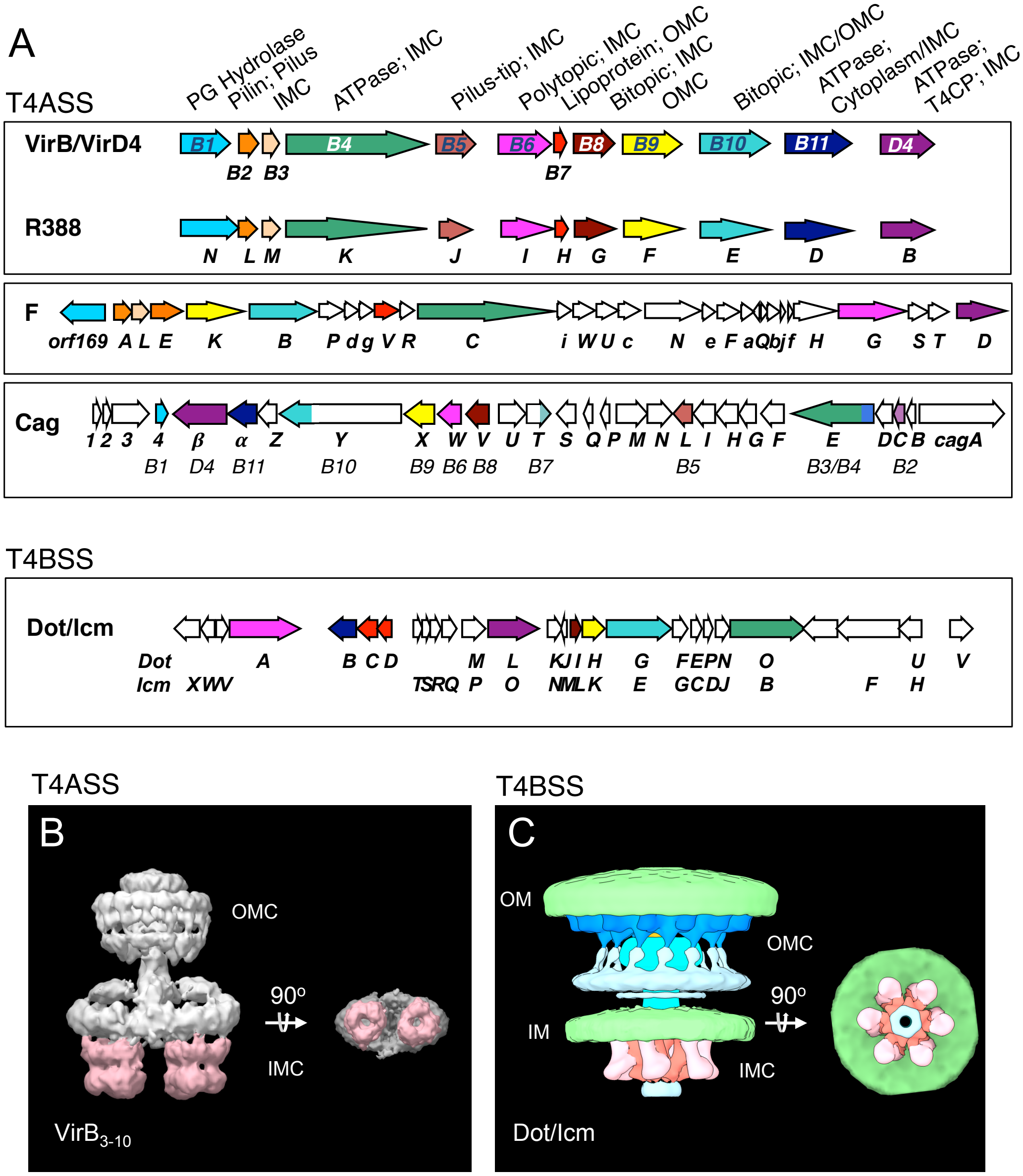

Fig. S1. Structures of T4SSs solved to date. A) Operon arrangements of type IV secretion systems (T4SSs) structurally analyzed to date. The T4SSs from gram-negative bacteria have been classified as T4ASS and T4BSS based on phylogenetic lineages. The VirB/VirD4 and R388-encoded transfer systems are representatives of 'minimized' T4ASSs. These systems are assembled from 'signature' subunits of T4SSs, including the 11 VirB subunits and the VirD4 substrate receptor or type IV coupling protein (T4CP). Functions and locations of machine subunits in the encoded T4SS are depicted at the top. The *Escherichia coli* F plasmid-encoded Tra system, and the *Helicobacter pylori* Cag T4SS are representatives of T4ASSs composed of 'signature' VirB/VirD4 subunits plus a number of other subunits or domains specific to those systems. The *L. pneumophila* Dot/Icm is a representative T4BSS also assembled from 'signature' subunits (color-coded) plus 15 or more system-specific subunits (no shading). **B)** Side and bottom views at 90° angles of the $VirB_{3\text{-}10}$ substructure, isolated from the R388 system and reconstructed by single-particle negative-stain electron microscopy **(EMD-2567),** showing the two side-by-side hexameric barrels of the VirB4 ATPase (pink-shaded). **C)** Side and bottom views at 90° angles of the *L. pneumophila* Dot/Icm machine obtained by *in situ* CryoET (EMD-7611, EMD-7612) showing the central hexamer of dimers of VirB4-like DotO (light/dark pink-shaded) and docked VirB11-like DotB (blue-shaded).
