# Supplementary material for: In Situ Molecular Architecture of the Helicobacter pylori Cag Type IV Secretion System
Source: mBio. 2019 May 14;10(3):e00849-19. doi: 10.1128/mBio.00849-19 (PMC6520456; doi:10.1128/mBio.00849-19)
Supplement: FIG S2 [file mBio.00849-19-sf002.pdf]

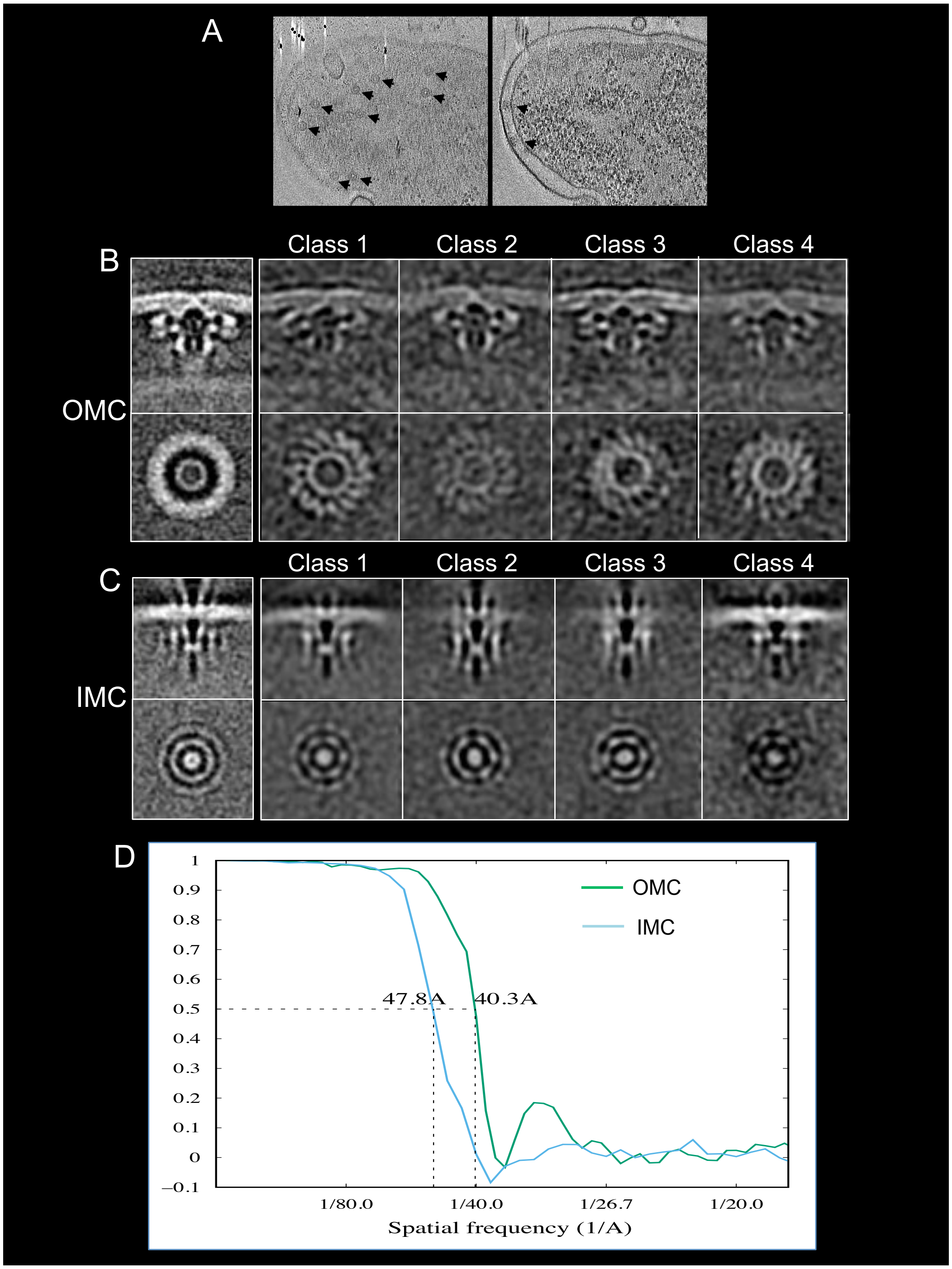

Fig. S2. Detection of the Cag$_{T4SS}$ on the *H. pylori* cell surface and refinements of the OMC and IMC. **A)** Multiple ring-shaped Cag complexes are detected on the cell surface (left) and spanning the periplasm (right), marked by black arrows. **B & C)** 3-D classifications reveal 14-fold symmetric features of the outer membrane complex (OMC) and 6-fold symmetric features of the inner membrane complex (IMC). For both the OMC and IMC, after the initial alignments, multivariate statistical analysis and hierarchical ascendant classification were used to generate four class averages. **B)** Left: A central section of the OMC and cross section view of the 3D average from the initial alignment. Right: Cross-sections from the OMC of four class averages showing 14-fold symmetries. **C)** Left: A central section of the IMC and cross section view of the 3D average from the initial alignment. Right: Cross-sections from the IMC of four class averages showing 6-fold symmetries. **D)** Fourier shell correlation plot of sub-volume averages aligned on the OMC (green) and IMC (blue) regions.
