# Supplementary material for: In Situ Molecular Architecture of the Helicobacter pylori Cag Type IV Secretion System
Source: mBio. 2019 May 14;10(3):e00849-19. doi: 10.1128/mBio.00849-19 (PMC6520456; doi:10.1128/mBio.00849-19)
Supplement: TABLE S1 [file mBio.00849-19-st001.pdf]

Table S1. Strains, plasmids, and CryoET data used in this study

H. pylori strain	Description	Antibiotic resistance	Reference
26695 wild-type	Intact *cag*PAI	None	(1)
Δ*cag* PAI	Deletion of *cag*PAI	Chloramphenicol	(1)
Δ*cagα*	Unmarked *cagα* deletion	Metronidazole	This study
Δ*cagβ*	Unmarked *cagβ* deletion	Metronidazole	This study
Δ*cagE*	Unmarked *cagE* deletion	Metronidazole	This study
Δ*cagX*	Unmarked *cagX* deletion	Streptomycin	(2)
Δ*cagY*	Unmarked *cagY* deletion	Streptomycin	(3)
Δ*cagT*	Unmarked *cagT* deletion	Metronidazole	(3)
Δ*cag3*	Unmarked *cag3* deletion	Streptomycin	(2)
Δ*cagM*	Unmarked *cagM* deletion	Metronidazole	(2)

Plasmid	Description	Antibiotic resistance	Reference
pΔ*cagα*	Contains sequences from HP0524 and HP0526, and deletion of HP0525 (*cagα)*	Ampicillin	This study
pΔ*cagβ*	Contains sequences from HP0523 and HP0525, and deletion of HP0524 (*cagβ)*	Ampicillin	This study
pΔ*cagE*	Contains sequences from HP0543 and HP0545, and deletion of HP0544 (*cagE*)	Ampicillin	This study
pΔ*cagα::cat-rdxA*	*cat-rdxA* cassette cloned into BamHI site in HP0525 locus of Δ*cagα* plasmid	Ampicillin, Chloramphenicol	This study
pΔ*cagβ::cat-rdxA*	*cat-rdxA* cassette cloned into BamHI site in HP0524 locus of Δ*cagβ* plasmid	Ampicillin, Chloramphenicol	This study
pΔ*cagE::cat-rdxA*	*cat-rdxA* cassette cloned into BamHI site in HP0544 locus of Δ*cagE* plasmid	Ampicillin, Chloramphenicol	This study

**Cryo-ET data**				
**No.**	**Strain**	**Tomograms**	**Machine particles**	**OMC?**
1	26695 (WT)	425	1280	Yes
2	Δ*cag*PAI	200	none visualized	No
3	Δ*cagβ*	666	2278	Yes
4	Δ*cagα*	419	1135	Yes
5	Δ*cagE*	242	465	Yes
6	Δ*cagX*	50	none visualized	No
7	Δ*cagY*	50	none visualized	No
8	Δ*cagM*	50	none visualized	No
9	Δ*cagT*	400	visualized	cylinders
10	Δ*cag3*	172	367	cylinders/knobs

References

1. **Busler VJ, Torres VJ, McClain MS, Tirado O, Friedman DB, Cover TL.** 2006. Protein-protein interactions among *Helicobacter pylori* Cag proteins. J Bacteriol **188:**4787-4800.
2. **Johnson EM, Gaddy JA, Voss BJ, Hennig EE, Cover TL.** 2014. Genes required for assembly of pili associated with the *Helicobacter pylori cag* type IV secretion system. Infect Immun **82:**3457-3470.
3. **Frick-Cheng AE, Pyburn TM, Voss BJ, McDonald WH, Ohi MD, Cover TL.** 2016. Molecular and structural analysis of the *Helicobacter pylori* Cag type IV secretion system core complex. MBio **7:**e02001-02015.
