# Supplementary material for: In Situ Molecular Architecture of the Helicobacter pylori Cag Type IV Secretion System
Source: mBio. 2019 May 14;10(3):e00849-19. doi: 10.1128/mBio.00849-19 (PMC6520456; doi:10.1128/mBio.00849-19)
Supplement: FIG S3 [file mBio.00849-19-sf003.pdf]

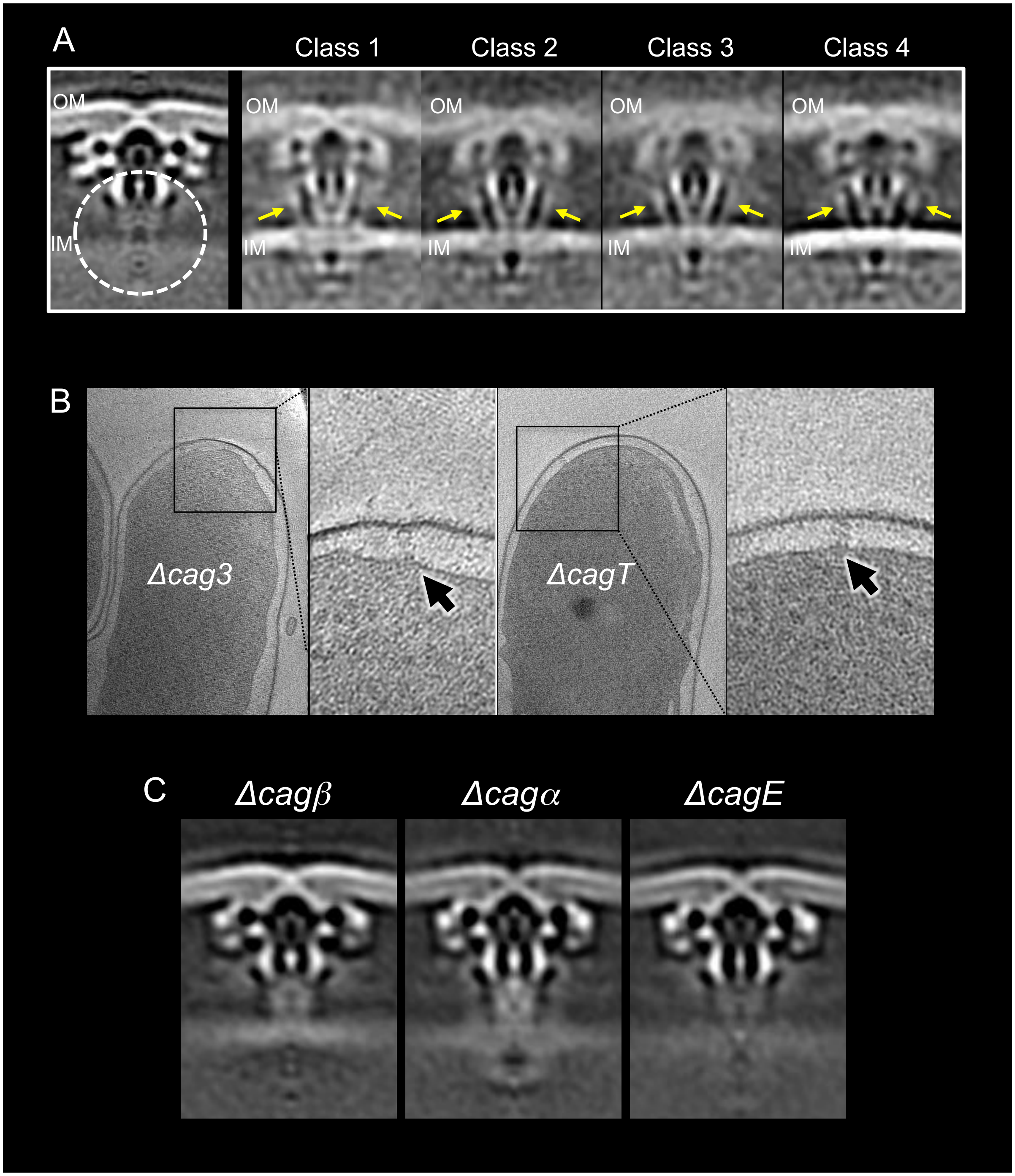

Fig. S3. Refinement of the native Cag_{T4SS} and visualization of Δ*cag3* and Δ*cagT* mutant machines. A) Local refinement of the periplasmic and IM-spanning portions of the Cag_{T4SS} boxed in the left panel. After the initial alignments, multivariate statistical analysis and hierarchical ascendant classification were used to generate four class averages. Left: A cross section view of the 3D average from the initial alignment. Right: Cross-sections of four class averages of the region within the dashed circle showing the central cylinder and surrounding collar, plus densities spanning the IM and extending into the cytoplasm. Arrows denote densities corresponding to the collar; however, due to apparent flexibility in the collar we were unable to generate a structure of this portion of the Cag_{T4SS}. **B)** Cag complexes detected on the surfaces of Δ*cag3* and Δ*cagT* mutants. A low-resolution structure was obtained for the Δ*cag3* mutant machine (see Fig. 1E,F), but the Δ*cagT* mutant machines were too few in number and morphologically variable to generate a structure by *in situ* CryoET. **C)** OMCs visualized in mutants deleted of the ATPases.
