# Supplementary material for: In Situ Molecular Architecture of the Helicobacter pylori Cag Type IV Secretion System
Source: mBio. 2019 May 14;10(3):e00849-19. doi: 10.1128/mBio.00849-19 (PMC6520456; doi:10.1128/mBio.00849-19)
Supplement: FIG S4 [file mBio.00849-19-sf004.pdf]

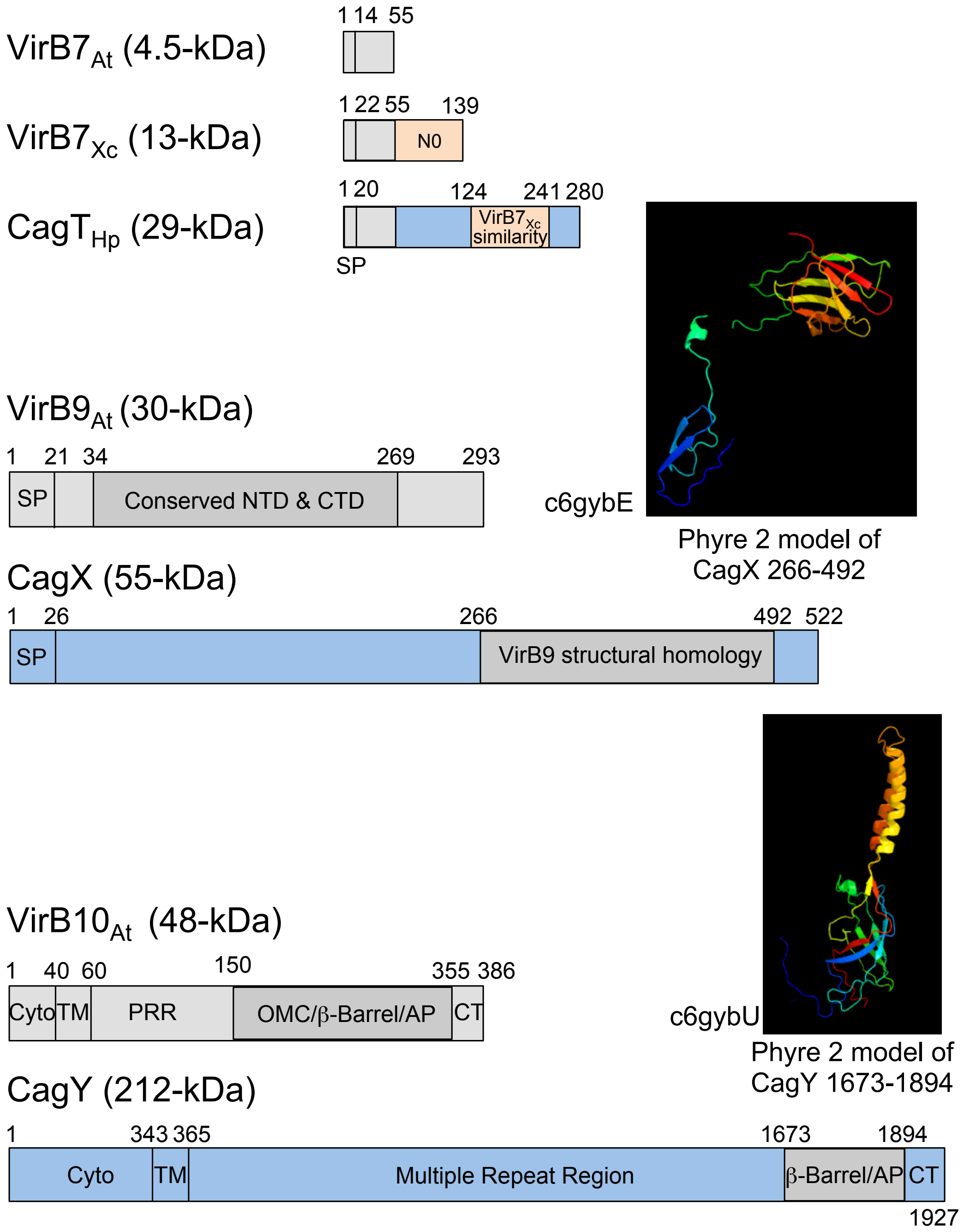

Fig. S4. Sequence and structural comparisons of VirB7, VirB9, and VirB10 components. Primary sequence organizations of the *A. tumefaciens* VirB proteins, the *X. citri* VirB7 subunit, and *H. pylori* Cag subunits. Molecular sizes (in kDa) are in parentheses. *A. tumefaciens* VirB subunits are in gray, and regions in the Cag orthologs with sequence or predicted structural similarity to the VirB subunits are similarly color-coded. $VirB7_{Xc}$ and the C-terminal region of CagT are related in primary sequence, although CagT is not predicted to fold as an N0 domain. Right: VirB9- and VirB10-like regions of CagX and CagY, respectively, adopt the structural folds of their homologs as deduced from Phyre2 modeling (1). Protein ID numbers of the closest structural templates, which in both cases are the VirB9 and VirB10 subunits of the *X. citri* T4SS, are listed. SP, signal peptide; NTD & CTD, N-terminal and C-terminal domains; TM, transmembrane domain; PRR, proline-rich region; OMC, outer membrane complex; AP, antennae projection; CT, C-terminal domain.

References

1. **Kelley LA, Mezulis S, Yates CM, Wass MN, Sternberg MJ.** 2015. The Phyre2 web portal for protein modeling, prediction and analysis. Nat Protoc **10:**845-858.
