# Supplementary material for: In Situ Molecular Architecture of the Helicobacter pylori Cag Type IV Secretion System
Source: mBio. 2019 May 14;10(3):e00849-19. doi: 10.1128/mBio.00849-19 (PMC6520456; doi:10.1128/mBio.00849-19)
Supplement: FIG S5 [file mBio.00849-19-sf005.pdf]

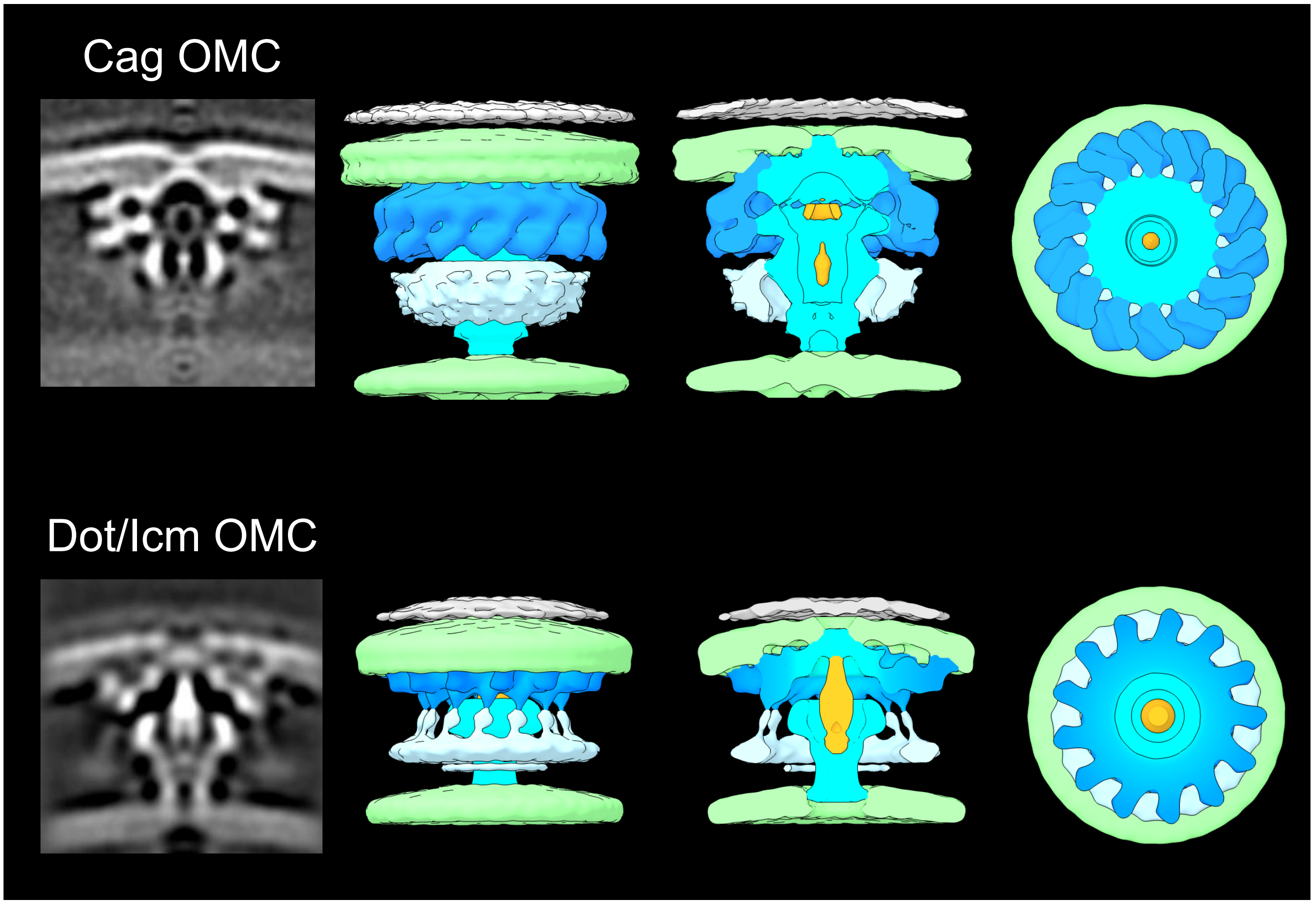

Fig. S5. Outer membrane complexes of the *H. pylori* Cag and *L. pneumophila* Dot/Icm T4SSs. Left: Central sections of subtomogram averages of the Cag and Dot/Icm OMCs. Right: 3D surface renderings of the Cag and Dot/Icm OMCs showing the spoked-wheel, collar, cylinder, and plug domains in side, central cut, and top-down views. The top-down views at the right show the counterclockwise rotation of the Cag OMC I-layer, and the clockwise rotation of the corresponding region of Dot/Icm OMC.
