# Supplementary material for: In Situ Molecular Architecture of the Helicobacter pylori Cag Type IV Secretion System
Source: mBio. 2019 May 14;10(3):e00849-19. doi: 10.1128/mBio.00849-19 (PMC6520456; doi:10.1128/mBio.00849-19)
Supplement: FIG S6 [file mBio.00849-19-sf006.pdf]

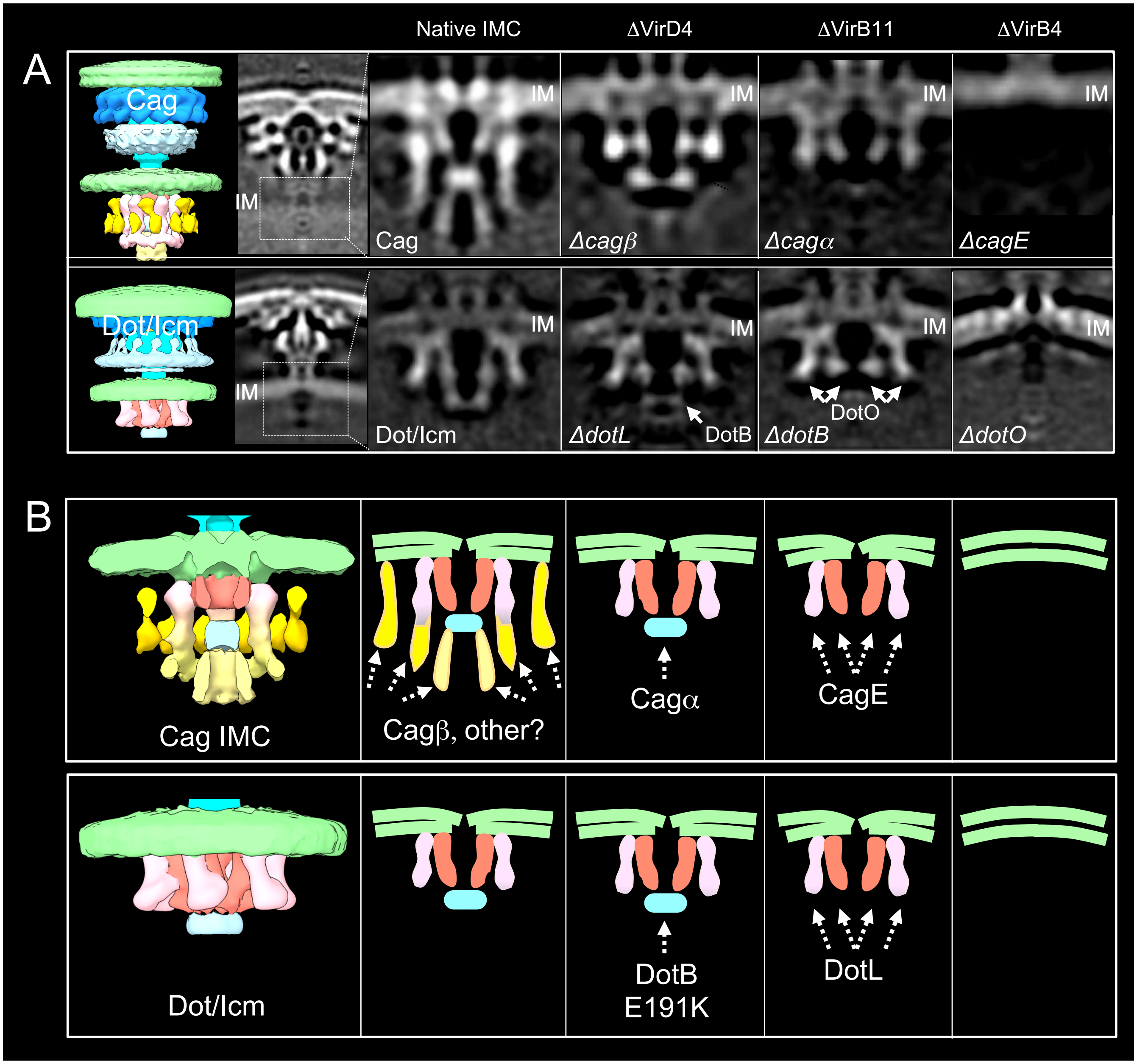

Fig. S6. Cytoplasmic complexes of the *L. pneumophila* Dot/Icm and *H. pylori* Cag T4SSs. A) Left: 3D renderings and central sections of subtomogram averages of the intact *L. pneumophila* Dot/Icm and Cag T4SSs, and local refinements of the IMCs (boxed regions). **ΔVirD4)** Mutant machines lacking the VirD4-like Cagβ and DotL substrate receptors. The Δ*cagβ* mutant machine is devoid of outer tiers of density, and the Δ*dotL* mutant machine resembles the native machine. The central disc of the native and Δ*dotL* machines is a DotB homohexamer. **ΔVirB11)** Mutant machines lacking the VirB11-like Cagα and DotB ATPases. Both machines are devoid of the central disc, but retain the side-by-side inverted V structures shown to consist of DotO in the Dot/Icm machine. **ΔVirB4)** Mutant machines lacking the VirB4-like CagE and DotO ATPases are devoid of all cytoplasmic densities. **B)** Cartoon schematics of the native and mutant machines showing densities contributed by the VirD4-, VirB11-, and VirB4-like subunits of the Cag and Dot/Icm systems. In the Dot/Icm system, DotB cycles on and off the IMC, necessitating use of a catalytically-dead variant (E191K) for visualization of the DotB central disc.
